# Intestinal Parasites in Children up to 14 Years Old Hospitalized with Diarrhea in Mozambique, 2014–2019

**DOI:** 10.3390/pathogens11030353

**Published:** 2022-03-14

**Authors:** Ofélia Luís Nhambirre, Idalécia Cossa-Moiane, Adilson Fernando Loforte Bauhofer, Assucênio Chissaque, Maria Luisa Lobo, Olga Matos, Nilsa de Deus

**Affiliations:** 1 Group of Opportunistic Protozoa/HIV and Other Protozoa, Global Health and Tropical Medicine, Medical Parasitology Unit, Instituto de Higiene e Medicina Tropical (IHMT), Universidade Nova de Lisboa, 1349-008 Lisboa, Portugal; luisalc@ihmt.unl.pt (M.L.L.); omatos@ihmt.unl.pt (O.M.); 2 Instituto Nacional de Saúde (INS), EN1, Bairro da Vila-Parcela n°3943, Distrito de Marracuene, Maputo 264, Mozambique; idaleciacossa@yahoo.com.br (I.C.-M.); adilsonbauhofer@gmail.com (A.F.L.B.); assucenyoo@gmail.com (A.C.); ndeus1@yahoo.com (N.d.D.); 3 Institute of Tropical Medicine, 2000 Antwerp, Belgium; 4 Instituto de Higiene e Medicina Tropical, Universidade Nova de Lisboa, 1349-008 Lisboa, Portugal; 5 Environmental Health Institute, Faculdade de Medicina da Universidade de Lisboa, 1649-028 Lisboa, Portugal; 6 Departamento de Ciências Biológicas, Universidade Eduardo Mondlane, Maputo 3453, Mozambique

**Keywords:** diarrhea, intestinal parasites, children, Mozambique

## Abstract

Diarrhea remains a public health problem in Mozambique, even with control strategies being implemented. This analysis aimed to determine the proportion and factors associated with intestinal parasitic infection (IPI) in children up to 14 years old with diarrheal disease, in the southern, central and northern regions of Mozambique. A single diarrheal sample of 1424 children was collected in hospitals and examined using the formol-ether concentration and modified Ziehl–Neelsen techniques to identify intestinal parasites using optical microscopy. Sociodemographic characteristics were obtained by questionnaires. Descriptive statistics and cross-tabulation were performed, and *p*-values <0.05 were considered statistically significant. A single IPI was detected in 19.2% (273/1424) of the children. *Cryptosporidium* spp. was the most common parasite (8.1%; 115/1424). Polyparasitism was seen in 26.0% (71/273), with the co-infection of *Ascaris lumbricoides* and *Trichuris trichiura* (26.8%; 19/71) being the most common. Age and province were related to IPI (*p*-value < 0.05). The highest occurrence of IPI was observed in the wet period (October to March), with 21.9% (140/640), compared to the dry period (April to September), with 16.9% (131/776) (*p*-value = 0.017). *Cryptosporidium* spp. and the combination of *A*. *lumbricoides/T*. *trichiura* were the main intestinal parasites observed in children hospitalized with diarrhea in Mozambique.

## 1. Introduction

In 2019, approximately half a million of the 5,050,000 total child deaths were due to diarrheal diseases and 53% of those deaths occurred in Sub-Saharan Africa, with a contribution of 7573 from Mozambique [1].

In Mozambique, diarrhea remains a public health problem even though strategies have been implemented since 1990, including the introduction of vaccination against rotavirus, national health week where children are vaccinated, dewormed and supplemented with vitamin A, and the improvement of water, sanitation and hygiene [2]. According to the United Nations Children’s Fund (UNICEF), Mozambique has about 320 daily deaths in children under five with malaria, respiratory infections or diarrhea [3]. Children aged between 6 and 23 months (19%) are more likely to have diarrhea compared to children under 6 months (5%) and children between 48 and 59 months (6%) in Mozambique [4].

Infectious diarrhea can be caused by multiple agents including viruses, bacteria, fungi and parasites. Among the parasites the main reported ones are *Entamoeba histolytica*, *Giardia duodenalis*, *Cryptosporidium* spp., *Ascaris lumbricoides*, *Ancylostoma/Necator* spp. and *Trichuris trichiura* [5]. Despite their worldwide distribution, *Enterobius vermicularis* [6] and *Dientamoeba fragilis* [7] have largely remained sub-diagnosed because of the limitations of the stool parasite screening methods commonly used. The symptoms and consequences of intestinal parasitic infections (IPI) include abdominal pain, intestinal obstruction, anaemia, poor appetite, worsening of nutritional status, impaired physical development and diarrhea [8].

Studies carried out in Mozambique in children up to 14 years old using microscopy between 2000 and 2013 revealed a wide range of parasites [9,10,11,12,13]. A national study conducted in school-age children (August 2005–June 2007) identified a great frequency of *A*. *lumbricoides*, *T*. *trichiura* and *Entamoeba coli*. *Enterobius vermicularis* was the fourth most common helminth found [9]. At a rural hospital in southern Mozambique, *A*. *lumbricoides*, *G*. *duodenalis* and *Strongyloides stercoralis* were the most frequent parasites [11]. In the south, Fonseca et al. [10] identified higher frequencies of *G*. *duodenalis*, *T*. *trichiura* and *A*. *lumbricoides* in the Hospital Central de Maputo (February and March 2009). In Sofala, one of the central provinces in Mozambique (June and August 2007), Meurs et al. [12] reported *T*. *trichiura*, *A*. *lumbricoides* and *S*. *stercoralis* as the most common parasites, while in the northern region of the country, in Nampula (2012 and 2013), Ferreira et al. [13] found *G*. *duodenalis*, *S*. *stercoralis* and *Cryptosporidium* spp. to be the most common; however, for *G*. *duodenalis* diagnosis, an Immunochromatographic assay was also applied in malnourished and HIV-positive children.

Among the protozoans, *Dientamoeba fragilis* was reported in Nampula Province in the northern region of the country in school-age children [14].

Except for a study developed nationwide in 2009, all other studies were conducted mainly in the southern region of the country. Other studies have been restricted to certain age groups (under five years old) or limited to children with some co-morbidities (HIV, malnutrition), and in some cases were carried out in children without suggestive symptoms. In this perspective, we used the data from the National Surveillance of Diarrhea (ViNaDia), implemented in 2014, with the aim of determining the burden and aetiology in children admitted with diarrhea. In this analysis, we aimed to describe the epidemiology of parasites in children up to 14 years of age in four of the eleven provinces of Mozambique, using a simple and inexpensive method—optical microscopy.

In addition, our data provide information regarding the variation in the distribution of intestinal parasites between 2014 and 2019.

## 2. Results

### 2.1. Characteristics of the Participants

Of the 2420 recruited children, 1424 provided a sufficient faecal sample for the screening techniques. Males accounted for 58.3% (830/1424) of these children, with 47.9% (682/1424) being below 12 months of age and 52.3% (745/1424) from Maputo (Table 1).

### 2.2. Intestinal Parasite Infections

Intestinal parasites were identified in 19.2% (273/1424; 95% CI: 17.2–21.3) and multiple intestinal parasite infections were detected in 26.0% (71/273; 95% CI: 21.1–31.4) of the children participating in the study.

**Figure 1:**
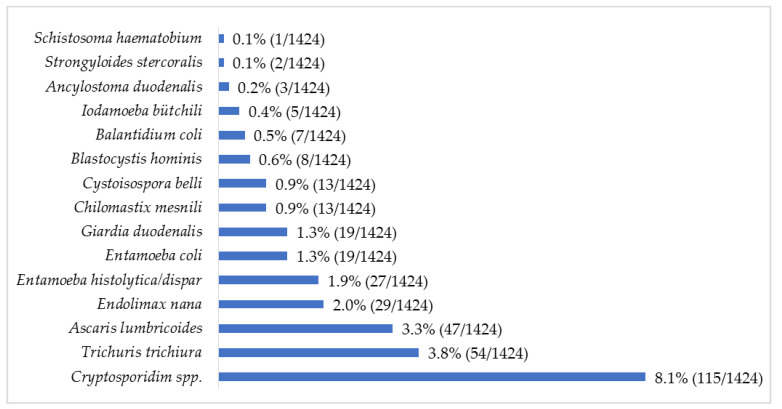
Relative frequency (%) of intestinal parasites in children with diarrhea in Mozambique, May 2014 to December 2019.

**Figure 2:**
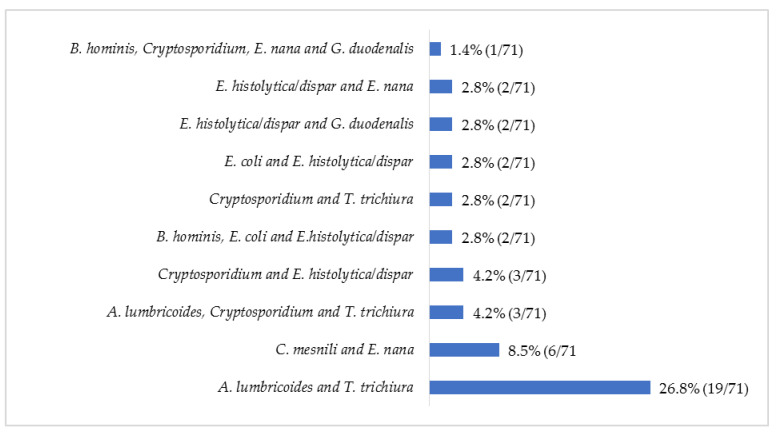
Relative frequency (%) of the most common multiple parasitic infections in children with diarrhea, May 2014 to December 2019.

The protozoan *Cryptosporidium* spp. was the most common parasite (8.1%; 115/1424) observed, followed by the helminth *T*. *trichiura* (3.8%; 54/1424) (Figure 1). Up to 39 combinations of multiple parasitic infections were recorded, ranging from two to four parasites (Figure 2). The most common parasitic combination was between the helminths *A*. *lumbricoides* and *T*. *trichiura* (26.8%; 19/71).

### 2.3. Factors Associated with Intestinal Parasite Infections

Age and province were related to IPI (*p*-value < 0.05), which was more common in children older than five years (30.8%; 20/65) and in children from Maputo (23.9%; 178/745) (Table 2). Multiple parasitic infections were more common in the provinces of Maputo (33.7%; 60/178) and Zambézia (28.6%; 6/21), followed by Nampula (3.6%; 2/56) and Sofala (16.7%; 3/18) (*p*-value < 0.001; Table 2).

### 2.4. Parasitic Distribution by Provinces

**Figure 3:**
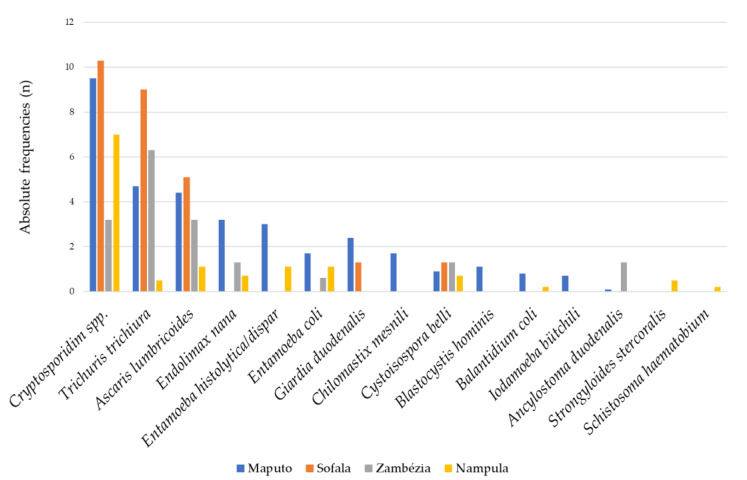
Relative frequency (%) of individual intestinal parasitic infections by province in children with diarrhea in Mozambique, May 2014 to December 2019.

The province of Sofala had the highest frequencies of the three most common parasites observed in the study: *Cryptosporidium* spp. 10.3% (8/78), *T*. *trichiura* 9.0% (7/78) and *A*. *lumbricoides* 5.1% (4/78). Despite this finding, all other parasites, with the exception of *G*. *duodenalis* and *Cystoisospora belli*, were not observed in this province, contrary to Maputo, which presented the highest parasite distribution (Figure 3). In Nampula Province, no *G*. *duodenalis* infections were diagnosed, but it was the only province where *S*. *stercoralis* was diagnosed (Figure 3).

### 2.5. Evaluation of the Distribution of Intestinal Parasites during the Wet and Dry Periods

The overall frequency of parasites observed during the rainy period was 21.9% (140/640; 95% CI: 18.8–25.2) and during the dry period it was 16.9% (131/776; 95% CI: 14.4–19.6) (*p*-value = 0.017).

The peaks of infection by *Cryptosporidium* spp. were mostly observed in the wet period during the study by 10.0% (64/640; 95% CI: 7.9–12.5), compared to 6.6% in the dry periods (51/776; 95% CI: 5.0–8.5; *p*-value = 0.019).

**Figure 4:**
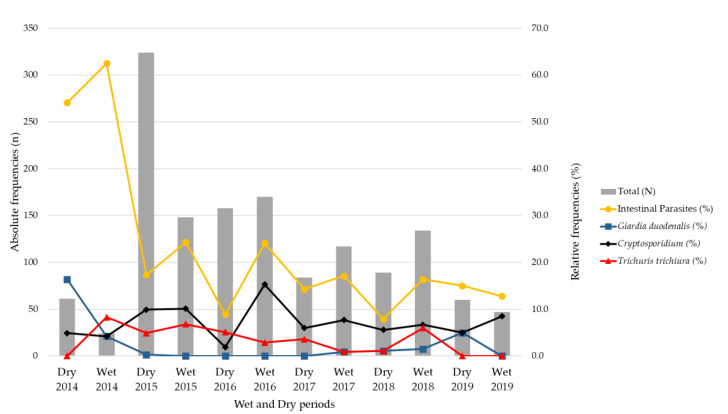
Relative frequency (%) of intestinal parasitic infections and the most frequent parasites (*Cryptosporidium* spp., *Trichuris trichiura* and *Giardia duodenalis)* by wet and dry period in children with diarrhea in Mozambique, May 2014 to December 2019.

*G*. *duodenalis* was more common in the dry period, with 1.9% (15/776; 95% CI: 1.1–3.1), and was less observed in the wet period, at 0.6% (4/640; 95% CI: 0.2–1.5); *p*-value = 0.033). Regarding *T*. *trichiura*, there were no significant differences in infection between the wet and dry periods (*p*-value = 0.657; Figure 4).

## 3. Discussion

One in five children with diarrhea was infected with at least one intestinal parasite. This finding was lower than that found in Nampula, which was 31.6% [13], and higher than that found in Hospital Central de Maputo, at 16.1% [10]. These differences can be explained by the different testing methods applied, causes of hospitalization and health unit level, inclusion criteria and possibly the local epidemiological conditions. A significant increase in infection was observed in children from Maputo and in those older than 59 months. An increase in exposure to the environment (soil, water, animals) associated with inadequate hygiene practices can help explain this result in this age group compared to the younger ones. It has also been proven that the youngest (breastfeeding children) are protected by natural immunological mechanisms decreasing the incidence as well as the duration of diarrhea [15]. Maputo was also the province with the greatest parasitic variability observed. This outcome may be due to the fact that this province had more hospitals involved in the study compared to the other provinces, along with a greater number of children tested.

In Mozambique, school starts at age six and the current strategy to control parasitic infections is focused on the school-age population. The Ministry of Health has been following WHO guidelines for preventive chemotherapy in schools since 2009 [16]. According to a geospatial analysis of the prevalence and intensity of soil-transmitted helminth infections in children, Sub-Saharan Africa has levels above the 2% threshold of the estimated prevalence of moderate-to-heavy infection. From this perspective, it is necessary to reinforce intervention to better target preventive chemotherapy to children at high risk of morbidity in order to monitor progress towards the soil-transmitted helminth targets for 2020 and 2030 [17].

One study carried out recently in Maputo markets reported that 29.3% of the horticultural products sampled presented at least one parasite (pathogenic and non-pathogenic) [18]. Therefore, it is also necessary to reinforce the improvement of sanitation and encourage healthy behaviours to avoid reinfections.

Protozoa were the most common group of parasites found, with an emphasis on *Cryptosporidium* spp., *G*. *duodenalis*, *E*. *histolytica/dispar*, *Cystoisospora belli* and *Balantidium coli* for being pathogenic. Even at low frequencies, pathogenic helminth infections (*T*. *trichiura*, *A*. *lumbricoides*, *A*. *duodenalis* and *S*. *stercoralis*) were recorded; these have been documented as predictors of growth retardation and impaired cognitive development, anaemia and vitamin A deficiency [19].

*Cryptosporidium* spp. was the most common parasite detected. This protozoon is known to cause diarrhea and malnutrition in young children in developing countries, and is associated with diarrhea outbreaks. In the study carried out by the Global Enteric Multicentre Study (GEMS) in the south of Mozambique, *Cryptosporidium* spp. was found to be the second most important cause of diarrhea [20]. In two hospitals in Maputo city, by microscopy, *Cryptosporidium* spp. was observed in 11% of the children sampled with diarrhea [21]. Its high prevalence can be justified by its opportunistic nature mainly in a country where the rates of HIV infection and malnutrition are higher [22]. *G*. *duodenalis* was less frequent than reported by other studies [10,13,23,24]. One of the possible reasons is the methods used, especially the immunological ones, which detect cyst epitopes, as opposed to the parasitological methods used by us, which only identify intact cysts and trophozoites. Additionally, the biology of the vegetative forms of *G*. *duodenalis*, the trophozoites, with the consistency of the stool samples collected may also have influenced the result. Trophozoites are considered to be the most common forms in watery and newly emitted stool; they are therefore not very resistant to the external environment [25]. The stool samples from our study were collected from children with diarrhea, and with the exception of Maputo, samples from the remaining provinces were sent to Maputo once a week and frozen, which probably destroyed the trophozoites and may have resulted in false negatives.

In this analysis we also observed that polyparasitism was prevalent, with the combination of *A*. *lumbricoides* and *T*. *trichiura* being the most common. *A*. *lumbricoides* was also reported as the most prevalent agent in a study conducted in South Africa, a country neighbouring Mozambique [26]. The occurrence of polyparasitism in this analysis may be due to the environmental contamination (poor sanitation, water supply provided), bad hygiene practices (poor hand washing, inadequate disposal of waste) as well as poverty. Individuals harbouring co-infections are usually at higher risk of significant morbidity than those infected with only one parasite species [27].

Mozambique has a tropical climate, with two periods: a cold-dry/winter period and a wet/summer period [28]. The wet periods are characterized by continuous heavy rains with flooding of residential and peri-urban areas. Our data, in general, indicate that parasitic infections were more common in wet periods. Among the analysed parasites, *Cryptosporidium* showed a significant seasonal pattern. These results are in agreement with studies in Kenya [29] and Nampula [30]. *Cryptosporidium* infection cases may be associated with floods and accidental ingestion of infectious forms, contamination of drinking water, unsafe treatment of sewage water or even the recycling of sewage for agriculture—situations that occur more often during the wet periods. *Giardia duodenalis* was common in dry periods, which contrasts with the study conducted in Nampula [30]. In Ghana, no significant association was found [31]. Although there was an association, between the dry period and giardiasis in this study, their interpretation must be performed carefully, given the smaller positive sample size found in the current study.

Our study had limitations, such as the lack of knowledge of the deworming status of the recruited children and the collection of one stool sample, which may have underestimated the real rate of infection by parasite once intestinal protozoa are irregularly shed [13,32]. Another limitation is related with the fact that samples from Sofala, Zambézia and Nampula were frozen before being sent to Maputo, where the analyses were carried out, a process which could have destroyed the diagnostic forms of the parasites. It is also important to mention the non-use of a cellulose tape slide test, a specific collection technique for *Enterobius vermicularis*, and the non-use of trichrome staining or modified iron-haematoxylin for *Dientamoeba fragilis*, which contributed significantly to its non-diagnosis and report in this study.

## 4. Materials and Methods

### 4.1. Design and Study Area

A cross-sectional hospital-based surveillance in the three regions of Mozambique was conducted in six health facilities from the National Diarrhea Surveillance (ViNaDia): in the southern region, Hospital Central de Maputo, Hospital Geral José Macamo and Hospital Geral de Mavalane in Maputo; in the centre, the Hospital Central da Beira in Sofala and the Hospital Geral de Quelimane in Zambézia and in the northern region, the Hospital Central de Nampula in Nampula. The sampling was carried out from May 2014 to December 2019, taking into account the cold/dry periods that occur from April to September and the wet season from October to March [28].

### 4.2. Population and Laboratory Procedures

The study population included 2420 children aged up to 14 years with diarrhea whose guardians had provided informed consent. Of these children, 1424 provided a sufficient faecal sample and were included in the present analysis. Children with nosocomial diarrhea were not eligible. Diarrhea was defined as the passage of three or more loose or liquid stools per day [33]. Sociodemographic data were obtained through a questionnaire administered by the health technicians to the caregivers or legal guardians of the participants.

A single stool sample was collected from each participant in a screw-capped container properly identified. The samples collected in Maputo were kept at 2 °C to 8 °C and sent on the same day to the laboratory of parasitology of the National Institute of Health/Instituto Nacional de Saúde–Moçambique (INS). The samples from the central and northern regions were kept at −20 °C and sent to the INS once a week. After delivery to the INS, the samples were subjected to the formalin-ether concentration technique for identification of intestinal parasites in general and to the modified Ziehl–Neelsen technique specifically for the identification of coccidian by optical microscopy [34]

Briefly, the presence of trophozoites, eggs and larvae of the parasites was identified by the formol-ether technique as described by the World Health Organization (WHO). The formol-ether technique consists in using 1 g of stool suspended in 10% formalin and ether and centrifuging it at 2500 rotation per minute. After centrifugation, the bottom was later used for observation in an optic microscopy after adding Lugol’s iodine solution. Parasites were observed in objective 10× and then changed to 40×.

The diagnosis of coccidian oocysts in stools was performed using the modified Ziehl–Neelsen stain method as described by the WHO [34]. This technique was performed by adding two smears in the slide, one from the fresh stool and the second from the formol-ether sediment. The smears were fixed with methanol and stained by adding a fuchsin solution, followed by an alcohol–acid solution. Finally, malachite green solution was added, and the preparation examined under the optical microscope at objective 100×.

### 4.3. Data Analysis

The data were analysed using the Statistical Package for the Social Sciences, Armok, NY, USA: IBM Corp, 2011, version 26.0 (IBM SPSS). All data collected were entered twice by two independent data entry typists and compared to evaluate consistency, prior analyses. Descriptive statistics were used to summarize population characteristics. Proportions and corresponding Jeffrey’s 95% confidence intervals (CI) were estimated for each infection and multiple infections. Bivariate analyses between dependent variables (infection by an intestinal parasite or infection by multiple parasites) and independent variables (sex, age and province) were conducted. Pearson’s Chi-square test or, if the assumptions were not met, the alternative Fisher’s exact test was used to identify factors related to the dependent variables, and the Mann–Whitney U test was used when the independent variable was quantitative. *p*-values < 0.05 were considered statistically significant.

## 5. Conclusions

In the present study, one in five children was infected with at least a parasite, and of these, one in four presented co-infection with more than one parasite. Pathogenic intestinal parasites were found to be circulating in the Mozambican paediatric population, with *Cryptosporidium* spp. and *T*. *trichiura* being the most common. Age was associated with IPI, and children in Maputo had the highest prevalence and the greatest parasite diversity. In evaluating some studies carried out in the country, it can be concluded that, in spite the effort of the governmental authorities, the frequency of IPI in Mozambique has remained unchanged and continues to be an indicator of poor hygiene and sanitation.

From this perspective, we recommend improving basic sanitation and runoff from rainwater. Awareness and education about individual and community hygiene practices with a special focus on kindergartens and schools will help to create a healthier society.

## Figures and Tables

**Table 1:** Demographic characteristics of children hospitalized with diarrhea in Mozambique, May 2014 to December 2019.

Characteristics	%	N = 1424
Gender		
Male	58.3	830
Female	41.7	594
Age in months (median([Q1–Q3]; min–max))	12 ((8–19); 0–168)	
Age categorized (in months)		
0–11	47.9	682
12–23	33.8	481
24–59	13.8	196
60–168	4.6	65
Province		
Maputo	52.3	745
Sofala	5.5	78
Zambézia	11.1	158
Nampula	31.1	443

N: Total number of samples tested; %: percentage/relative frequency.

**Table 2:** Cross-tabulation between sociodemographic factors and intestinal parasitic infections in children with diarrhea in Mozambique, May 2014 to December 2019.

Global Intestinal Parasite Infection (N = 1424)	%	n/N	*p*-Value
Gender			0.340 ^a^
Male	18.3	152/830	
Female	20.4	121/594	
Age in months among positives (median ([Q1-Q3]; min–max))	13 ((9–20); 2–150)		0.019 ^b^
Age in months among negatives (median ([Q1-Q3]; min–max))	12 ((8–19); 0–168)		
Age categorized (in months)			0.042 ^a^
0–11	17.0	116/682	
12–23	20.2	97/481	
24–59	20.4	40/196	
60–168	30.8	20/65	
Province			<0.001 ^a^
Maputo	23.9	178/745	
Sofala	23.1	18/78	
Zambézia	13.3	21/158	
Nampula	12.6	56/443	
Multiple intestinal infections (N = 273)			
Gender			0.124 ^a^
Male	22.4	34/152	
Female	30.6	37/121	
Age in months among positives (median ([Q1-Q3]; min–max))	14 ((9–24); 2–136)		0.791 ^b^
Age in months among negatives (median ([Q1-Q3]; min–max))	13 ((9–20); 4–15)		
Age categorized (in months)			0.508 ^a^
0–11	25.9	30/116	
12–23	23.7	23/97	
24–59	35.0	14/40	
60–168	20.0	4/20	
Province			<0.001 ^c^
Maputo	33.7	60/178	
Sofala	16.7	3/18	
Zambézia	28.6	6/21	
Nampula	3.6	2/56	

N: Total number of samples tested; %: percentage/relative frequency; ^a^ Chi-square test; ^b^ Mann–Whitney U test; ^c^ Fisher’s exact test.

## Data Availability

The data presented in this study are available upon request from the corresponding author.
